# Lateral Kangaroo Care in Hemodynamic Stability of Extremely Preterm Infants: Protocol Study for a Non-Inferiority Randomized Controlled Trial CANGULAT

**DOI:** 10.3390/ijerph19010293

**Published:** 2021-12-28

**Authors:** Laura Collados-Gómez, Laura Esteban-Gonzalo, Candelas López-López, Lucía Jiménez-Fernández, Salvador Piris-Borregas, Esther García-García, Juan Carlos Fernández-Gonzalo, Esther Martínez-Miguel

**Affiliations:** 1 Faculty of Biomedicine, Nursing and Nutrition Department, Universidad Europea de Madrid, 28670 Madrid, Spain; laura.collados@universidadeuropea.es (L.C.-G.); ESTHER.GARCIA@universidadeuropea.es (E.G.-G.); JUANCARLOS.FERNANDEZ@universidadeuropea.es (J.C.F.-G.); esther.martinez@universidadeuropea.es (E.M.-M.); 2 Department of Neonatal Intensive Care, Hospital Universitario 12 de Octubre, (H12O), 28041 Madrid, Spain; candelas.lopez@salud.madrid.org (C.L.-L.); jimenez.lucia@gmail.com (L.J.-F.); piris.salvador@gmail.com (S.P.-B.); 3 Care Research Group (Invecuid), Instituto de Investigación Sanitaria 12 de Octubre (imas12), 28041 Madrid, Spain; 4 Faculty of Nursing, Physiotherapy and Podiatry, Nursing Department, Universidad Complutense de Madrid, 28040 Madrid, Spain; 5 Researcher at the Maternity and Childhood Health Research Group (Area 4), 12 de Octubre University Hospital, 28041 Madrid, Spain

**Keywords:** kangaroo mother care, preterm infant, temperature, neonatal pain, intraventricular hemorrhage, nursing

## Abstract

Introduction: This study aims to assess the efficacy of the modified kangaroo care lateral position on the thermal stability of preterm neonates versus conventional kangaroo care prone position. Material and methods: A non-inferiority randomized parallel clinical trial. Kangaroo care will be performed in a lateral position for the experimental group and in a prone position for the control group preterm. The study will take place at the neonatal intensive care unit (NICU) of a University Hospital. The participants will be extremely premature infants (under 28 weeks of gestational age) along the first five days of life, hemodynamically stable, with mother or father willing to do kangaroo care and give their written consent to participate in the study. The sample size calculated was 35 participants in each group. When the premature infant is hemodynamically stable and one of the parents stays in the NICU, the patient will be randomized into two groups: an experimental group or a control group. The primary outcome is premature infant axillary temperature. Neonatal pain level and intraventricular hemorrhage are secondary outcomes. Discussion: There is no scientific evidence on modified kangaroo care lateral position. Furthermore, there is little evidence of increased intraventricular hemorrhage association with the lateral head position necessary in conventional or prone kangaroo care in extremely premature newborns. Kangaroo care is a priority intervention in neonatal units increasing the time of use more and more, making postural changes necessary to optimize comfort and minimize risks with kangaroo care lateral position as an alternative to conventional prone position kangaroo care. Meanwhile, it is essential to ensure that the conventional kangaroo care prone position, which requires the head to lay sideways, is a safe position in terms of preventing intraventricular hemorrhage in the first five days of life of children under 28 weeks of gestational age. Trial registration at clinicaltrials.gov: NCT03990116.

## 1. Introduction

Kangaroo care (KC) is a safe and low-cost intervention with an impact on the prevention of many potential complications in neonates and premature infants such as sepsis, brain injury, or mortality [1]. Conventional KC consists of placing the baby vertically between the mother’s breasts or on the father’s thorax, skin-to-skin with their limbs flexed, and in the prone position with the head 90 degrees to the side and slightly extended to ensure proper opening of the airway and to favor visual contact between the mother/father and child [2].

Plentiful evidence is available on the benefits of prone kangaroo care, or skin-to-skin, such as lower mortality (RR 0.64; 95% [CI] 0.46, 0.89), reduced risk of neonatal sepsis (RR 0.53, 95% CI 0.34, 0.83), of hypothermia (RR 0.22; 95% CI 0.12, 0.41), of hypoglycemia (RR 0.12; 95% CI 0.05, 0.32), and of hospital readmissions (RR 0.42; 95% CI 0.23, 0.76) as well as greater hemodynamic stability (lower respiratory rate, greater peripheral oxygen saturation). KC increases the likelihood of exclusive breastfeeding (RR 1.50; 95% CI 1.26, 1.78), and neonates experience less pain, better sleep organization, and more attachment, with better cognitive development and emotional regulation in the first year of life [3,4,5,6,7].

Two of the main complications in premature infants are hypothermia due to increasing heat loss and germinal matrix intraventricular hemorrhage (IVH).

Hypothermia is a major complication observed in extreme prematures due to thermoregulatory center’s physiological immaturity. They also have a greater relationship between the body surface and total body mass, vasomotor control immaturity and limited subcutaneous fat. The skin is also porous, not covered by lanugo or vernix caseosa, and has a thin corneal stratum. Prematures are poikilotherm, having a body temperature that varies according to the outside temperature, so they need greater environmental control, including the temperature and the humidity [8].

Temperature is an outcome that increases the risk of morbidity and mortality in this population. Mohamed et al. [9] found the hypothermia is associated with mortality (OR = 1.89; 1.72–2.09), intra-ventricular hemorrhage (OR = 1.86; 1.09–3.14), bronchopulmonary dysplasia (OR = 1.28; 1.16–1.40) or neonatal sepsis (OR = 1.47; 1.09–2.49), and retinopathy of prematurity (OR = 1.45; 1.28–1.72). Similar results were found in other studies [10]. Laptook et al. [11] found in an adjusted analysis that with each 1 °C decrease in body temperature, sepsis and mortality risks increase by 11 and 28%, respectively.

The ILCOR (International Liaison Committee on Resuscitation) [12] has insisted on the importance of maintaining normothermia (36.5–37.5 °C), especially in more premature infants given that, even though it is rarely the primary cause of death, it is related to increased morbidity and mortality rates and impact of complications such as IVH, bronchopulmonary dysplasia, retinopathy of prematurity, and increased rate of neonatal sepsis or necrotizing enterocolitis [13]. Recommendations to keep up normothermia are high humidity concentrations in incubators (above 80%), use of polyethylene bags or thermal mattresses, and skin-to-skin care [14,15]). Abundant literature is available on hypothermia in premature infants and the effectiveness of KC to prevent it; however, few studies have been carried out in the most vulnerable neonatal population, extremely premature infants [16,17].

However, the greater risk of hypothermia is taking place during neonatal transfer, which is defined as the movement from incubator to kangaroo position on father or mother chest. The reason why this happens is that neonates get in contact with an environment not as controlled as the incubator ones in terms of temperature and humidity. Furthermore, during kangaroo care, parents provide heat to their premature child but not humidity, another reason why the risk of hypothermia increase [18].

Meanwhile, the rate of IVH stands at around 20–38% in children under 28 weeks of gestational age (GA) and is more frequent in the first 72 h of life [19,20]. Thus, the risk of bleeding is inversely related to gestational age and can be attributed to three factors: vascular immaturity of the germinal matrix, deficient extravascular matrix, and cerebral blood flow alterations. In conventional kangaroo care prone position to a premature infant, the head is turned 90 degrees, compressing the internal jugular vein and therefore compromising venous blood drainage. This potential venous congestion leads to increased intracranial pressure, reduced brain oxygenation, and ultimately of the germinal matrix, thus increasing the risk of IVH [21]

KC is a neuroprotective intervention that can improve survival in premature infants [14], becoming the star program at neonatal units around the world; this is the reason to recommend it as soon as it is possible, and for as long time, requiring postural changes of the neonate towards the KC lateral position if KC is prolonged [22,23].

Although some studies have proposed that premature infants with lateral head position have poorer cerebral vascular flow, cerebral oximetry, and greater risk of IVH, evidence of this is scarce and low quality [24]. Thus, recommendations in clinical practice are variable; on occasions, preterms under 28 weeks of GA should be positioned with the head in the midline position for at least the first 72 h and up to the first seven days of life, and with the incubator inclined 15–30 degrees [24,25,26]. This would entail placing extremely premature in the modified KC lateral position during the first 7 days of life, lying on the bare thorax or chest of the mother or father, with the limbs flexed towards the midline and the head neutral and in line with the rest of the body. However, there is no evidence of the effectiveness of modified KC lateral position in relation to thermal. One side lying position could be a thermoregulation challenge, compared with prone position, because there is less skin surface in contact with the skin of the kangaroo provider.

There is no evidence either on hemodynamic stability and the consequences on pain or stress during one side lying position KC.

Meanwhile, clinical practice is also variable in terms of recommending the conventional KC prone position in neonates with an umbilical catheter as it cannot be visually controlled and therefore possible related complications monitored (displacement, bleeding, leakage), with no scientific evidence confirming a greater risk of these adverse events. However, these restrictive clinical practices are found in published studies [27].

## 2. Material and Methods

### 2.1. Aim

The main aim of this study is to assess the efficacy of the modified kangaroo care lateral position on the thermal stability of preterm infants versus conventional kangaroo care prone position. Secondary proposed aims are (a) to compare the number of intraventricular hemorrhage events in relation to the assigned group, prone or lateral position; and (b) to assess the effectiveness of kangaroo care lateral position in alleviating pain versus conventional prone kangaroo care.

### 2.2. Design

The study is designed as a non-inferiority randomized parallel clinical trial.

The CONSORT guidelines for randomized controlled trials will be used. This protocol also adheres to the SPIRIT guidelines (Supplementary Materials: checklist SPIRIT).

### 2.3. Study Setting

The study will be conducted at the NICU of a University Hospital in Madrid, Spain. This NICU is a level-IIIC and a 24/7 open doors for parents. The NICU has 19 beds, with 9 single-family rooms (two rooms are twins) and 8 beds in an open bay and family-centered and developmentally supportive care are implemented in it. Parents are invited to participate and be part of their childcare, as is indicated by the evidence [28]. From the admission, parents work together with staff. The NICU has implemented a standardized parental training program called “Cuidame” (“take care of me”) which teaches parents how to participate gradually.

On the other hand, when preterm birth is expected, parents are informed about different treatment options by the neonatal team. This information includes how important it is to stay next to babies’ incubators, be part of their childcare, and provide early kangaroo care as soon as baby condition permits it. If it is possible, parents can also visit the unit before being admitted.

### 2.4. Study Population

The participants of the study will be all patients admitted to the NICU premature under 28 weeks of GA hemodynamic stability, with a parent (mother or father) who wants to do KC and give their written consent to participate in the study.

The exclusion criteria will be premature infant (a) receiving high-frequency ventilation; (b) in the immediate recovery period from major surgery; (c) with abdominal wall defect; (d) who needs restraints; (e) with hemodynamically unstable; or (f) mother/father with physically or psychologically incapacitating illness.

Nowadays, there is no scientific consensus about the definition “hemodynamically unstable”. For that reason, the research team chose to define it as repeated apneas followed by bradycardia (HR < 100 bpm) as a current participant condition or during handling if it requires an increase in oxygen demands or stimulation for restoring breathing.

Removal criteria: hypothermia or kangaroo care duration under 60 min.

### 2.5. Sample Size

The primary outcome was the neonatal body temperature difference between KC prone position and KC lateral position. The sample size was calculated to achieve an 80% statistical power with an α of 0.05 to detect a 0.3 °C difference in the body temperature (DS 0.16) [5,13], and the non-inferiority margin was set at a 0.2 difference in body temperature [13]. In addition, we estimated a 10% loss to follow-up, so the sample size calculated is 35 participants in each group.

Additionally, the sample size was calculated to the secondary outcome; it was the difference in neonatal pain level between KC prone position and KC lateral position. With the same power an α to detect 0.83 points (DS 1), the non-inferiority margin was set at 1.53 points in PIPP-R, and we estimated a 10% loss, the sample size calculated is 29 participants in each group [5].

Given the scarce and heterogenous literature available on IVH [24], the populational behavior of this variable is unknown, with no references to calculate the sample size.

Therefore, the minimum estimated sample size to respond to the aims proposed is 35 subjects in each group.

### 2.6. Randomization and Allocation

Participants will be recruited through non-probabilistic consecutive sampling.

Potentially eligible newborn patients will be identified in the research staff’s daily clinical practice or referred to them for eligibility assessment once they have been identified by clinicians who are not research staff.

Premature infants who meet all of the inclusion criteria and will be performing kangaroo care for the first time will be assigned to the experimental or control group on a simple 1:1 randomized basis, using a list of randomized numbers according to ID assignment from 1 to 70, using the random.org website.

### 2.7. Blinding

Both clinicians/caregivers and research nurses will be informed of the group to which each participant is assigned due to their involvement in facilitating the care intervention for each group. However, non-caregiver researchers and those responsible for processing and analyzing the data will be blinded to assignment.

### 2.8. Intervention

**Figure 1:**
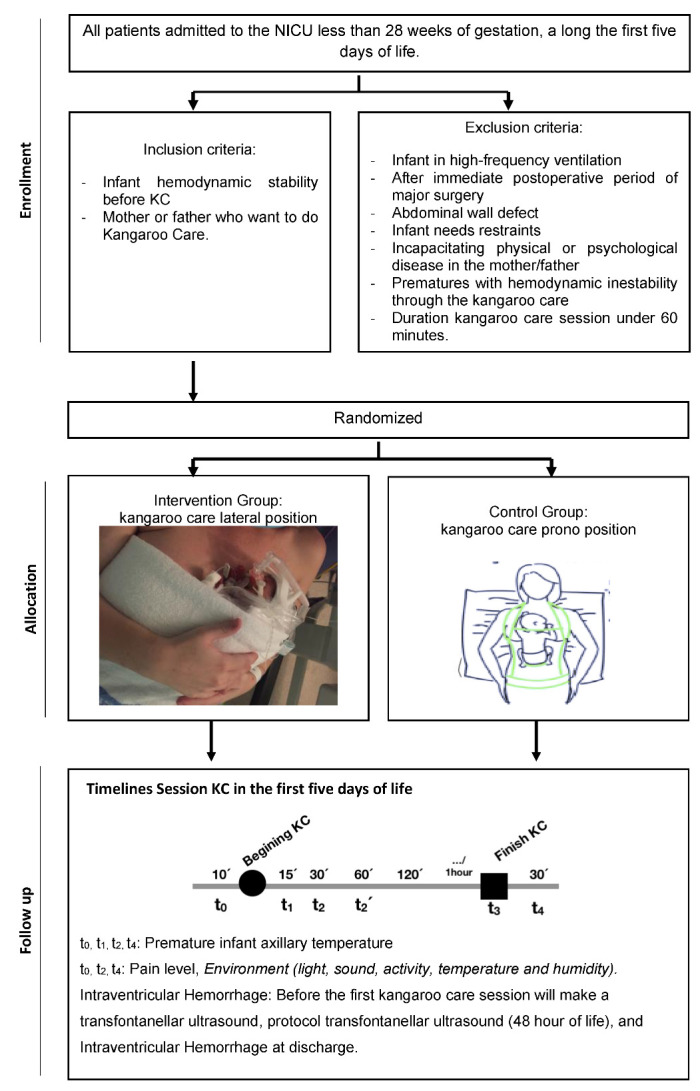
Flow of participants.

When the extremely premature infant is hemodynamically stable and one of the parents is staying at the NICU, the patient will be randomized into two groups: an experimental group or a control group. Said group allocation will be maintained along the first five days of life when KC will be performed (Figure 1. Flow of participants).

In the experimental group, KC lateral position will be performed. Premature infants will stay in between the mother’s breasts or father’s chest, in the upright position and side-lying, providing maximal skin-to-skin contact, with the limbs, arms, and legs bent to the midline. (Figure 1).

In the control group, KC prone position will be performed, so the baby will be placed in the upright position between the mother’s breasts or father’s chest and in a ventral position with its head to the side (90°), slightly extended to free the airway and to encourage visual contact between the mother/father and their child (Figure 1).

All infants in kangaroo care will be inside the polyethylene bags (NeoHelp TM^®^ (Vygon, Padova, Italy) to bring humidity into the environment and decrease hypothermia risk in the way we are reducing heat loss avoiding skin evaporation. The infant’s head will be covered, but keeping the skin-to-skin contact so that in the prone position, the bag will be open in the anterior side of the baby, and in the lateral position, it will be open in its lateral side.

The transfer will be done by the mother/father or the nurse. Nevertheless, during transfer, staff will always be present to ensure the safety, as well as the correct handling and fixing of lines, wires, and circuits, making sure everything is correct once the transfer is finished.

During KC sessions, we will provide comfort and privacy to the families. Parents will sit down in a reclinable armchair during KC. The study is carried out in a mixed allocation NICU where we have single-family rooms (with glass doors that allow staff to have a direct viewing from outside) and an open bay setting.

Participants in single-family rooms are monitored continuously through a central monitoring system which is controlled by NICU staff, alarms limits are set, and the direct view from outside is possible. Beyond this, parents can ring the bell in case they need to ask for help.

Premature infants’ axillary temperature will be assessed during kangaroo care. In the event of axillary temperature <36.5 °C, checks will be conducted to ensure the temperature sensor is placed correctly, adding a towel or blanket to the polyethylene bags covering it. The temperature will be reassessed after 15 min, adding another towel or blanket if it remains <36.5 °C. It will be assessed for the third time after 15 min, and kangaroo care will be suspended if the temperature remains <36.5 °C.

If during this time the neonate should have an axillary temperature >37.5 °C, the cover material (towel or blanket) will be gradually removed.

KC sessions can be at any time of the day, with the frequency and duration required by the mother/father, based on a minimum of 60 min. All KC sessions during the first five days of life will be monitored, recording data in the medical record, which will later be extracted and systematized in the study data collection notebook for analysis.

### 2.9. Participant Timeline

The participant timeline is presented in Table 1: participant timeline.

### 2.10. Outcomes

Primary outcome:

- Premature infant axillary temperature.

Secondary outcomes:

- Neonatal pain level.
- Intraventricular hemorrhage number of events.

### 2.11. Data Collection Methods

See Table 1: Participant timeline.

The following dependent variables will be obtained:

- Premature infant axillary temperature measured continuously with the temperature probe fixed (AirLife^TM^ Infant Skin temperature probe (Vyaire Medical Inc, Chicago, IL, USA), Giraffe^®^ incubator (GE Healthcare, Chicago, IL, USA) on the axillary infant with adhesive tape (protect the infant skin with hydrocolloid tape before).

The axillary temperature will also be taken with a Braun^®^ PRT2000 (Braun GmbH, Kronberg, Germany) thermometer.

Neonatal pain level assessed by Premature Infant Pain Profiled Revised. PIPP-R is a multidimensional scale (it includes physiological, behavioral, and facial expression signs for the pain assessment) developed by Stevens B [29], is valid for assessing pain in full-term and preterm neonates during painful procedures. Scores range from 0 to 21; score minor to 7 is interpreted as no pain or minor pain. Studies validating the scale show a high correlation between PIPP and PIPP-R is not painful (R2 = 0.91, *p* < 0.0001) and painful (R2 = 0.98, *p* < 0.0001) procedures. Average applicability scores are 3.5–3.7 (average of 4 out of 5) [30].

● Intraventricular hemorrhage (IVH) assessed by cranial ultrasonography using Siemens AcusonX300 ^®^ ultrasound system (Siemens AG, Berlin, Germany):
  ○ IVH presence: yes or no.
  ○ Grade [31]
    - Grade 1: hemorrhage located in the subependymal germinal matrix.
    - Grade 2: intraventricular blood content occupies less than 50% of the intraventricular area.
    - Grade 3: blood occupies >50% and distends ventricle.
    - Grade 4: significant overdistension of lateral ventricles and the entire ventricular system in general.
  ○ Location: right, left, or bilateral.
  ○ Date of cranial ultrasonography.

Transfontanelar ultrasound will be performed at two different times, the first before the first KC session by a specialist in neonatology with specific training in neonatal ultrasound, under early IVH detection screening. The head US done before the first KC session is used as a control US to identify the presence of congenital HIV or bleeding on the first hours of life. This way, we can determine it is not associated with the kangaroo position in prone.

The second ultrasound at 48 h of life (according to the study unit protocol) by a specialist in radiodiagnosis.

The following independent variables will be obtained from each participant in every KC session:

- Demographic variables: gestational age at birth, gender, and birth date.
- Clinical data: Apgar score (test to assess the extrauterine adaptation of the newborn 1, 5, and 10 min after birth), type of delivery (eutocic, instrumental, cesarean), birth weight, gestational age, actual weight, hours of life, pathology at five days of life, analgesia administered (drug, route, dose and time since the last dose), painful/stressful procedures during kangaroo session, respiratory support (support type and fraction of inspired oxygen).
- Hemodynamics-related: maximum heart rate and minimum peripheral oxygen saturation observed for 20 s, apneas. Continuous hemodynamic monitoring to measure the heart rate, peripheral oxygen saturation, and apnea with Philips IntelliVue MP60/MP70^®^ monitor (Koninklijke Philips Electronics N.V., Eindhoven, Netherland) or Masimo SET^®^ oximetry monitor (Masimo, Irvine, CA, USA).
- Temperature-related: Giraffe ^®^ (GE Healthcare, Chicago, IL, USA) incubator air temperature and humidity; temperature and humidity of room or open-bay setting, where neonate is located assessed by Multi-Function Environment Meter PCE EM882 (PCE, Durham, United Kingdom), and temperature of humidifier chamber Dräger VentStar^®^ (Dräger, Lubeca, Germany).
- Kangaroo care: position (prone or lateral), date of KC session, session number, duration, KC provider (mother/father), axillary temperature of KC provider, materials used for kangaroo care, person transferring neonate.
- Environment room or open-bay setting: light and sound in macroenvironment assessed by Multi-Function Environment Meter PCE EM882, and activity inside the box room or open-bay setting with scale NIDCAP Nursery Certification Program [32].
- Adverse events or incidents: accidental removal of the line, such as umbilical venous or arterial catheters, venous catheter, or endotracheal tubes.
- The long-term outcomes we have collected are the number of days in NICU stay, the total number of days in the hospital, the number of days on mechanical ventilation, and mortality.
- The patient’s medical history will be used for the collection of demographic, clinical, and intraventricular hemorrhage (IVH)-related data. And the direct observation for measuring pain, activity inside the room or open-bay setting, and rest of outcome. They will be extracted and transferred to the data collection notebook.

### 2.12. Data Management

The data obtained from the participants will be included in an SPSS database (version 25) (IBM, Chicago, IL, USA). Data will be entered by the lead researcher, who will be the only person with access to the database.

Patients will be coded according to the order in which they join the study, following number allocation, through randomization.

Prior to statistical analysis, in order to improve the quality of data collected, the database will be debugged with the summarize function, identifying outliers, and the analysis of losses.

### 2.13. Statistical Analysis

Normality associated with each quantitative variable will be analyzed using the test Kolmogorov–Smirnov test.

A descriptive analysis will be performed of the demographic and clinical characteristics of subjects in both treatment groups: quantitative variables will be described by mean or median and their dispersion measurements, standard deviation or interquartile range, respective, as appropriate. Qualitative variables will be described by absolute frequencies and percentages. The descriptive analysis will be accompanied by the 95% confidence interval.

Prior to testing hypotheses, we will check whether both groups are homogeneous in terms of the baseline characteristics, using Student’s *t*-test or Mann–Whitney U for quantitative variables or ANOVA or Kruskal–Wallis if the variable does not follow normal distribution, and if statistically significant differences are found, we will continue with post hoc Bonferroni contrasts or weighted comparisons with Mann–Whitney U. Chi-squared will be used for qualitative variables.

If the groups are heterogeneous, confounding factors will be defined to adapt the final analysis to the main variable.

The effectiveness of KC modified lateral position compared to the KC conventional prone position will be assessed, calculating axillary temperature variation (Student’s t-test or Mann–Whitney U), level of pain (Student’s *t*-test or Mann–Whitney U), and IVH development (Chi-squared) in both groups.

Intention to treat (NNT) and protocol analyses will be performed. Should the results be conflicting, protocol deviations and/or breaches will be analyzed.

Safety analysis in relation to adverse events will be calculated using Pearson’s Chi-squared test or Fisher’s exact test as appropriate.

An explanatory model with stepwise linear regression will be created based on a significance level of 0.1, and confounding variables and their level of importance will be determined.

The statistical significance level will be *p* < 0.05. Data will be analyzed with the Statistical Package for the Social Sciences-IBM SPSS^®^ program version 25.0 (IBM, Chicago, IL, USA).

### 2.14. Validity and Reliability/Rigour

The clinical trial offers guarantees in terms of internal validity, reducing possible bias and confounding variables. However, before data analysis, group homogeneity will be verified, and a multivariant analysis carried out to detect possible confounding and/or interaction variables.

Prior to starting the study, researchers will agree the data collection notebook and prepare instructions. Before collecting data, a pilot of the data collection notebook will be run to validate its content and the homogeneity of the data collected. A short video tutorial on data collection will also be collected.

### 2.15. Monitoring

An interim analysis is performed on the primary endpoint when 20% of patients have been randomized. The idea is to be able to identify an increase in HIV incidence in the control group, such as other potential hazard events in all participants. In that case, the study would come to a halt. The main investigator will have unblinded access to data and will discuss the results of the interim analysis with the research team.

The trial will not be stopped in case of futility related to the outcome variables because kangaroo care is the current intervention in the neonatal units due to clinically proven benefits, so futility is not sustainable.

The main investigator has the ultimate authority to stop or modify the trial and to ensure the protocol’s continued. He will follow up with all participants, and he will check all kangaroo care sessions are completed as it is referred to in the protocol. If that is not the case, the session will be discarded.

Changes on the protocol will be notified to all team members immediately and keeping all documentation standardized. All updated versions will be available in a digital form, and team members will be noticed during practical and theoretical lectures.

Safety and complications were monitored by identifying kangaroo potential adverse events noted down in the Data Collection Logbook (DCL), such as hypothermia, continuous hemodynamic instability during kangaroo care, or accidental displacement of any therapeutic device. Those events will be collected and analyzed in the first 24 h by the investigator in charge of the session, notifying the main investigator. The main investigator will record this adverse event on the study database, taking the actions required according to the severity and impact.

A committee in charge of data monitoring has not been created, the tracking is carried out by the research team. Non-audits have been scheduled.

Preliminary analyses every 20 new participants will be done to discard a rising HIV incidence in the control group, which would mean the study would come to a standstill.

Established withdrawal criteria are hypothermia or a Kangaroo care session less than 60 min.

## 3. Discussion

As recommended in various societies [33], neonatal units must be designed taking into account the fundamental role of families, fostering the creation of a family room versus an open bay, obtaining friendlier, more intimate, and comfortable spaces for the family, incorporating neonatal care and promoting kangaroo care at any time, provided the neonate is stable. This requires validating alternative postures to the kangaroo care prone position as the baby will be in KC for long hours, and postural changes must be made to prevent possible complications as in traditional incubator care.

KC is endorsed by multiple studies, although scarce in the population of study of this paper, infants under 28 weeks of GA [17]. Furthermore, no studies have currently been found that analyze the effectiveness of alternative kangaroo positions other than prone kangaroo care, aiming to contribute evidence of the effectiveness of lateral position KC in terms of thermal stability and pain alleviation.

Meanwhile, we must determine the safety of the KC prone position in relation to increased intraventricular hemorrhage in premature under 28 weeks of GA during their first days of life, as evidence found is scarce and controversial [23].

The possible limitations of the study are as follows: (a) in terms of the study population, due to the immaturity of the premature infants, the sample size may be reached in a broad period of time caused as possible hemodynamic instability may not allow kangaroo care the first days of life; (b) regarding intervention, the first kangaroo care session may be delayed until approximately 20 h of life due to the numerous formalities required of the father, and as the mother is normally not available to act as kangaroo due to health issues as she is not admitted in the same room as the baby; (c) related to biased information as the data collection notebook is not fully filled in due to healthcare team workload, always guaranteeing the collection of dependent variables; (d) ultrasound sensitivity in early intraventricular hemorrhage detection; (e) the relatively small sample size for the IVH outcome. The sample size for this secondary outcome was not calculated due to the heterogenous literature available about IVH in relation to the risk that would be caused by the prone position compared with the other positions; and finally, (f) pain is measured on the validated PIPP-R scale but not validated in Spanish; there is currently no validated scale in Spanish for assessing neonatal pain. In addition, with regard to the assessment of pain, it will not be possible to blind the observers or to carry out the measurement of pain by two professionals independently.

These restrictions will be taken into account during data analysis, analyzing missing values, and, if necessary, completing simple imputation of these values.

## 4. Conclusions

This will be the first study contributing scientific evidence on modified kangaroo care lateral position as an alternative to conventional prone kangaroo care; we only found one study to evaluate the kangaroo supported diagonal flexion positioning on safety and early communication, but the sample size is limited, and the outcomes are different [34].

With greater parent participation in caring for their children in the NICU context, and based on scientific evidence, kangaroo care is ever more extended in frequency and duration, but neonate postural changes must be proposed, incorporating the practice of modified kangaroo care lateral position.

On the other hand, this study aims to provide evidence in relation to better positioning during the first days of life of extremely premature babies to prevent intraventricular hemorrhage related to neonate morbidity and mortality.

## Figures and Tables

**Table 1:** Participant timeline. SPIRIT Schedule of enrolment, interventions and assessment.

	Activity/Assessment	Staff Member	Approximate Time to Complete	Before First Session Kangaroo Care	t_0_	t_1_	t_2_	t_2_’	t_3_	t_4_	t_5_
enrollement	Informed consent	Study coordinator/study staff	10 min	x							
	Inclusion/exclusion form	Study coordinator/study staff	2 min	x							
	Allocation	Study coordinator/study staff	1 min	x							
Intervention	Intervention A: Prone kangaroo care	Neonatal nurse	5 min								
	Intervention B: Lateral Kangaroo care	Neonatal nurse	5 min								
	Eco-TF	physician	5–10 min	x							
Assessment	neonatal axillary temperature	Neonatal nurse	<1 min		x	x	x	x		x	
	Score pain	Neonatal nurse	min		x		x	x		x	
	Eco- TF: Intraventricular hemorrhage (IVH)	physician	5–10 min	x							x
	mother/father axillary temperature	Neonatal nurse	10 seg		x		x	x	x		
	Incubator temperature	Neonatal nurse	1.5 min		x					x	
	Incubator humidity	Neonatal nurse			x					x	
	Heater temperature	Neonatal nurse			x		x	x		x	
	Heart rate	Neonatal nurse			x		x	x		x	
	Oxygen Saturation	Neonatal nurse			x		x	x		x	
	Fio2	Neonatal nurse			x		x	x		x	
	Apnea				x		x	x		x	
	Box temperature	Neonatal nurse	1 min		x		x	x		x	
	Box humidity	Neonatal nurse			x		x	x		x	
	Sound	Neonatal nurse			x		x	x		x	
	Light	Neonatal nurse			x		x	x		x	
	Activity inside the box	Neonatal nurse	2 min		x		x	x		x	
	Duration kangaroo care	Neonatal nurse	<1 min								
	days in NICU stay	Study coordinator	5 min								x
	days in the hospital	Study coordinator	5 min								x
	days on mechanical ventilation	Study coordinator	5 min								x
	Mortality at 6 month old	Study coordinator	5 min								x

t_0_: 10 min previous the kangaroo care. t_1_: 15 min after beginning the kangaroo care. t_2_: 30 min after beginning the kangaroo care. t_2_’: if the duration kangaroo care is longer than 60 min, It assessment each 60 min after beginning the kangaroo care, so, 120 min after beginning the kangaroo care; 180 min after beginning the kangaroo care, etc. t_3_: at finish the kangaroo care. t_4_: 30 min after finish the kangaroo care. t_5_: delivery.

## Data Availability

Data not available due to privacy and copyright protection.

## Supplementary Materials

The following supporting information can be downloaded at: https://www.mdpi.com/article/10.3390/ijerph19010293/s1, Table S1: Scheme 2013. Checklist: Recommended items to address in a clinical trial protocol and related documents.

## Abbreviations

GA	Gestational age
IVH	Intraventricular hemorrhage
KC	Kangaroo care
NICU	Neonatal intensive care unit
NIDCAP	Newborn Individualized Developmental Care and Assessment
PIPP-R	Premature Infant Pain Profile-Revised
