# Supplementary material for: Lateral Kangaroo Care in Hemodynamic Stability of Extremely Preterm Infants: Protocol Study for a Non-Inferiority Randomized Controlled Trial CANGULAT
Source: Int J Environ Res Public Health. 2021 Dec 28;19(1):293. doi: 10.3390/ijerph19010293 (PMC8750690; doi:10.3390/ijerph19010293)
Supplement: Supplementary file 1 [file ijerph-19-00293-s001.zip › ijerph-1470997-supplementary.pdf]

SPIRIT

STANDARD PROTOCOL ITEMS: RECOMMENDATIONS FOR INTERVENTIONAL TRIALS

Scheme 2013. Checklist: Recommended items to address in a clinical trial protocol and related documents .

Section/Item	Item No	Description	Addressed on Page Number
Administrative information			
Title	1	Descriptive title identifying the study design, population, interventions, and, if applicable, trial acronym	1 (main text)
Trial registration	2a	Trial identifier and registry name. If not yet registered, name of intended registry	1 (main text); 2 (title page)
	2b	All items from the World Health Organization Trial Registration Data Set	______________
Protocol version	3	Date and version identifier	4 (main text)
Funding	4	Sources and types of financial, material, and other support	2 (title page)
Roles and responsibilities	5a	Names, affiliations, and roles of protocol contributors	1 (title page)
	5b	Name and contact information for the trial sponsor	______________
	5c	Role of study sponsor and funders, if any, in study design; collection, management, analysis, and interpretation of data; writing of the report; and the decision to submit the report for publication, including whether they will have ultimate authority over any of these activities	_____________
	5d	Composition, roles, and responsibilities of the coordinating center, steering committee, endpoint adjudication committee, data management team, and other individuals or groups overseeing the trial, if applicable (see Item 21a for data monitoring committee)	_____________
Introduction			
Background and rationale	6a	Description of research question and justification for undertaking the trial, including summary of relevant studies (published and unpublished) examining benefits and harms for each intervention	3–6 (main text)
	6b	Explanation for choice of comparators	3–6 (main text)
Objectives	7	Specific objectives or hypotheses	6 (main text)
Trial design	8	Description of trial design including type of trial (e.g., parallel group, crossover, factorial, single group), allocation	6–7 (main text)

		ratio, and framework (e.g., superiority, equivalence, non-inferiority, exploratory)	
Methods: Participants, interventions, and outcomes			
Study setting	9	Description of study settings (e.g., community clinic, academic hospital) and list of countries where data will be collected. Reference to where list of study sites can be obtained	7 (main text)
Eligibility criteria	10	Inclusion and exclusion criteria for participants. If applicable, eligibility criteria for study centers and individuals who will perform the interventions (e.g., surgeons, psychotherapists)	7 (main text)
Interventions	11a	Interventions for each group with sufficient detail to allow replication, including how and when they will be administered	8–11 (main text); Figure 1
	11b	Criteria for discontinuing or modifying allocated interventions for a given trial participant (e.g., drug dose change in response to harms, participant request, or improving/worsening disease)	__________
	11c	Strategies to improve adherence to intervention protocols, and any procedures for monitoring adherence (e.g., drug tablet return, laboratory tests)	__________
	11d	Relevant concomitant care and interventions that are permitted or prohibited during the trial	8–9 (main text)
Outcomes	12	Primary, secondary, and other outcomes, including the specific measurement variable (e.g., systolic blood pressure), analysis metric (e.g., change from baseline, final value, time to event), method of aggregation (e.g., median, proportion), and time point for each outcome. Explanation of the clinical relevance of chosen efficacy and harm outcomes is strongly recommended	11–5 (main text); Figure 2
Participant timeline	13	Time schedule of enrolment, interventions (including any run-ins and washouts), assessments, and visits for participants. A schematic diagram is highly recommended (see Figure)	Figure 2
Sample size	14	Estimated number of participants needed to achieve study objectives and how it was determined, including clinical and statistical assumptions supporting any sample size calculations	7–8 (main text)
Recruitment	15	Strategies for achieving adequate participant enrolment to reach target sample size	8 (main text)
Methods: Assignment of interventions (for controlled trials)			
Allocation:			

Sequence generation	16a	Method of generating the allocation sequence (e.g., computer-generated random numbers) and list of any factors for stratification. To reduce predictability of a random sequence, details of any planned restriction (e.g., blocking) should be provided in a separate document that is unavailable to those who enroll participants or assign interventions	8 (main text)
Allocation concealment mechanism	16b	Mechanism of implementing the allocation sequence (e.g., central telephone; sequentially numbered, opaque, sealed envelopes), describing any steps to conceal the sequence until interventions are assigned	8 (main text)
Implementation	16c	Who will generate the allocation sequence, who will enroll participants, and who will assign participants to interventions	8 (main text)
Blinding (masking)	17a	Who will be blinded after assignment to interventions (e.g., trial participants, care providers, outcome assessors, data analysts) and how	8 (main text)
	17b	If blinded, circumstances under which unblinding is permissible, and procedure for revealing a participant's allocated intervention during the trial	______
Methods: Data collection, management, and analysis			
Data collection methods	18a	Plans for assessment and collection of outcome, baseline, and other trial data, including any related processes to promote data quality (e.g., duplicate measurements, training of assessors) and a description of study instruments (e.g., questionnaires, laboratory tests) along with their reliability and validity, if known. Reference to where data collection forms can be found if not in the protocol	11–15 (main text)
	18b	Plans to promote participant retention and complete follow-up, including list of any outcome data to be collected for participants who discontinue or deviate from intervention protocols	______
Data management	19	Plans for data entry, coding, security, and storage, including any related processes to promote data quality (e.g., double data entry, range checks for data values). Reference to where details of data management procedures can be found if not in the protocol	15 (main text)
Statistical methods	20a	Statistical methods for analyzing primary and secondary outcomes. Reference to where other details of the statistical analysis plan can be found if not in the protocol	15–16 (main text)
	20b	Methods for any additional analyses (e.g., subgroup and adjusted analyses)	16 (main text)

	20c	Definition of analysis population relating to protocol non-adherence (e.g., as randomized analysis), and any statistical methods to handle missing data (e.g., multiple imputation)	16 (main text)
Methods: Monitoring			
Data monitoring	21a	Composition of data monitoring committee (DMC); summary of its role and reporting structure; statement of whether it is independent from the sponsor and competing interests; and reference to where further details about its charter can be found if not in the protocol. Alternatively, an explanation of why a DMC is not needed	17 (main text)
	21b	Description of any interim analyses and stopping guidelines, including who will have access to these interim results and make the final decision to terminate the trial	17 (main text)
Harms	22	Plans for collecting, assessing, reporting, and managing solicited and spontaneously reported adverse events and other unintended effects of trial interventions or trial conduct	17–18 (main text)
Auditing	23	Frequency and procedures for auditing trial conduct, if any, and whether the process will be independent from investigators and the sponsor	18 (main text)
Ethics and dissemination			
Research ethics approval	24	Plans for seeking research ethics committee/institutional review board (REC/IRB) approval	20 (main text)
Protocol amendments	25	Plans for communicating important protocol modifications (e.g., changes to eligibility criteria, outcomes, analyses) to relevant parties (e.g., investigators, REC/IRBs, trial participants, trial registries, journals, regulators)	____________
Consent or assent	26a	Who will obtain informed consent or assent from potential trial participants or authorized surrogates and how (see Item 32)	20 (main text)
	26b	Additional consent provisions for collection and use of participant data and biological specimens in ancillary studies, if applicable	____________
Confidentiality	27	How personal information about potential and enrolled participants will be collected, shared, and maintained in order to protect confidentiality before, during, and after the trial	15 (main text)
Declaration of interests	28	Financial and other competing interests for principal investigators for the overall trial and each study site	21
Access to data	29	Statement of who will have access to the final trial dataset, and disclosure of contractual agreements that limit such access for investigators	15 (main text)

Ancillary and post-trial care	30	Provisions, if any, for ancillary and post-trial care and for compensation to those who suffer harm from trial participation	____________
Dissemination policy	31a	Plans for investigators and sponsor to communicate trial results to participants, healthcare professionals, the public, and other relevant groups (e.g., via publication, reporting in results databases, or other data-sharing arrangements), including any publication restrictions	____________
	31b	Authorship eligibility guidelines and any intended use of professional writers	____________
	31c	Plans, if any, for granting public access to the full protocol, participant-level dataset, and statistical code	____________
Appendices			
Informed consent materials	32	Model consent form and other related documentation given to participants and authorized surrogates	____________
Biological specimens	33	Plans for collection, laboratory evaluation, and storage of biological specimens for genetic or molecular analysis in the current trial and for future use in ancillary studies, if applicable	____________
